# 5-Methyl-3,3-bis­(4-methyl­piperazin-1-yl)-1-[2-(4-methyl­piperazin-1-yl)eth­yl]indolin-2-one

**DOI:** 10.1107/S1600536812022416

**Published:** 2012-05-23

**Authors:** Hui-Hui Lin, Xiao-Lin Zheng, Sheng-Li Cao

**Affiliations:** a Department of Chemistry, Capital Normal University, Beijing 100048, People’s Republic of China

## Abstract

In the title compound, C_26_H_43_N_7_O, each piperazine ring adopts a chair conformation. Two 1-methyl­piperazine rings bond to one C*sp* ^3^ of the pyrrole ring *via* the piperazine N atoms, while the third one links to the N atom of the indolin-2-one unit through a flexible ethyl group with an almost *syn* conformation. In the crystal, mol­ecules are connected through methyl­ene–carbonyl C—H⋯O inter­actions into an infinite chain along the *c* axis. The almost parallel arrays are stacked, forming a three-dimensional framework.

## Related literature

For the background to indoline-2,3-dione and its derivatives, see Chiyanzu *et al.* (2005 ▶); Solomon *et al.* (2009 ▶); Sriram *et al.* (2004 ▶). For a related structure, see: Lin *et al.* (2012 ▶).
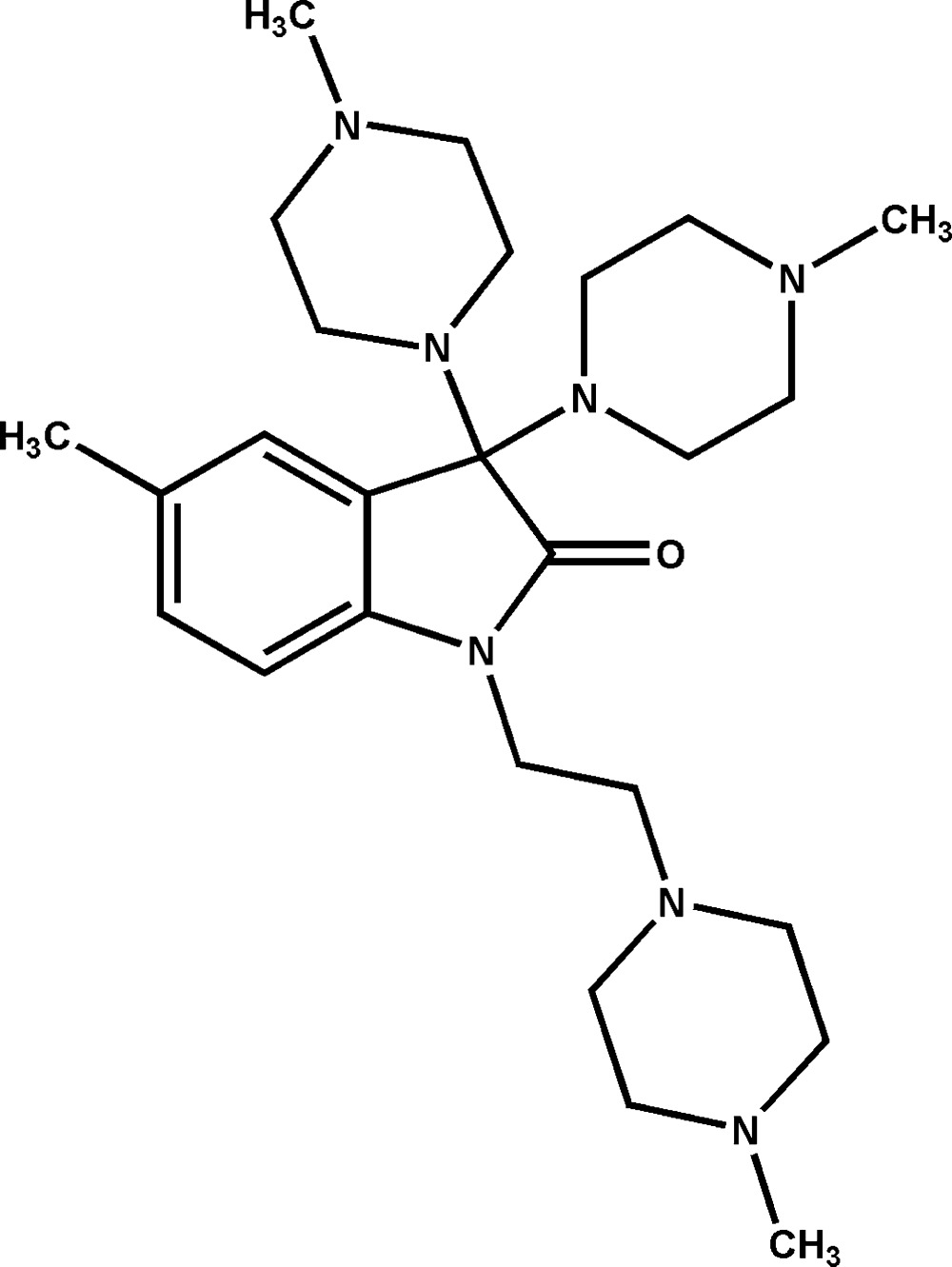


## Experimental

### 

#### Crystal data

C_26_H_43_N_7_O
*M* *_r_* = 469.67
Monoclinic, 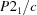

*a* = 15.9433 (8) Å
*b* = 14.4097 (6) Å
*c* = 12.5310 (6) Å
β = 108.774 (3)°
*V* = 2725.7 (2) Å^3^
*Z* = 4
Mo *K*α radiation
μ = 0.07 mm^−1^
*T* = 296 K
0.33 × 0.21 × 0.20 mm

#### Data collection

Bruker APEXII CCD area-detector diffractometer
Absorption correction: multi-scan (*SADABS*; Bruker, 2007 ▶) *T*_min_ = 0.613, *T*_max_ = 0.746
17378 measured reflections
4785 independent reflections
3332 reflections with *I* > 2σ(*I*)
*R*_int_ = 0.036

#### Refinement

*R*[*F* ^2^ > 2σ(*F* ^2^)] = 0.047
*wR*(*F* ^2^) = 0.129
*S* = 1.03
4785 reflections
307 parameters
H-atom parameters constrained
Δρ_max_ = 0.23 e Å^−3^
Δρ_min_ = −0.20 e Å^−3^

### 

Data collection: *APEX2* (Bruker, 2007 ▶); cell refinement: *APEX2* and *SAINT* (Bruker, 2007 ▶); data reduction: *SAINT*; program(s) used to solve structure: *SHELXS97* (Sheldrick, 2008 ▶); program(s) used to refine structure: *SHELXL97* (Sheldrick, 2008 ▶); molecular graphics: *SHELXTL* (Sheldrick, 2008 ▶); software used to prepare material for publication: *SHELXTL* and *PLATON* (Spek, 2009 ▶).

## Supplementary Material

Crystal structure: contains datablock(s) I, global. DOI: 10.1107/S1600536812022416/zj2076sup1.cif

Structure factors: contains datablock(s) I. DOI: 10.1107/S1600536812022416/zj2076Isup2.hkl

Supplementary material file. DOI: 10.1107/S1600536812022416/zj2076Isup3.cml

Additional supplementary materials:  crystallographic information; 3D view; checkCIF report

## Figures and Tables

**Table 1:** Hydrogen-bond geometry (Å, °)

*D*—H⋯*A*	*D*—H	H⋯*A*	*D*⋯*A*	*D*—H⋯*A*
C19—H19*B*⋯O1^i^	0.97	2.64	3.388 (2)	135

Symmetry code: (i) 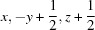.

**Fig. 1.**
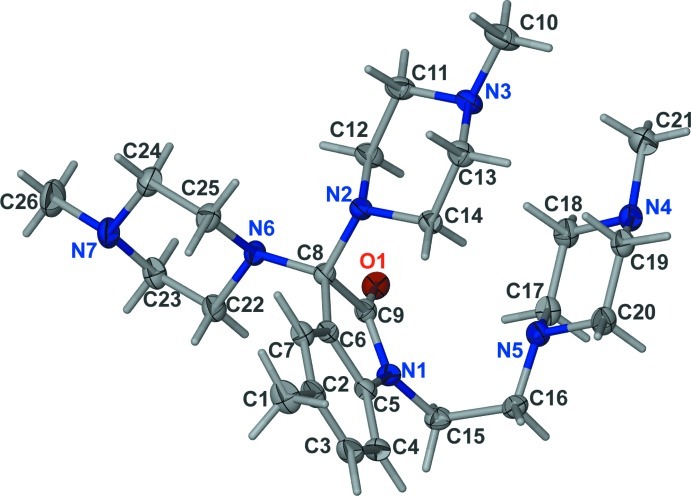
The title molecule with the atomic numbering scheme. The displacement ellipsoids of the non-hydrogen atoms are shown at the 30% probability level.

**Fig. 2.**
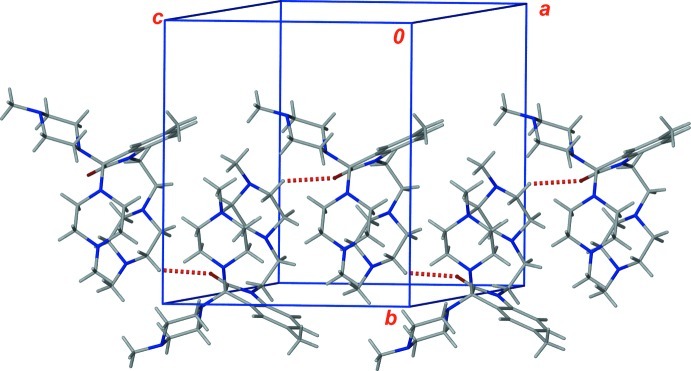
The array of the title compound along the c direction. The red-dashed lines indicate C19—H19b···O2i interactions (i x, -y + 0.5, z + 0.5).

**Fig. 3.**
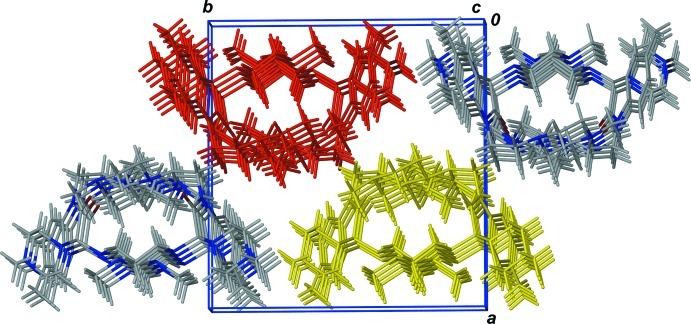
View down the c direction of the stacking structure of the title compound. The stacking structures are shown with different color for clarity.

## supplementary crystallographic information

### Comment

Mannich base derivatives of indoline-2,3-dione (isatin) exhibit antibacterial (Chiyanzu *et al.*, 2005), anti-HIV (Sriram *et al.*, 2004) and anticancer activity (Solomon *et al.*, 2009).Recently, we obtained an isatin derivative with an flexible ethylene linker between the N atom of the isatin moiety and the amine group of the morpholine through the reaction of 1-(2-bromoethyl)-5-methylindoline-2,3-dione with excess of morpholine, namely 5-methyl-3,3-bis(morpholin-4-yl)-1-[2(morpholin-4-yl)ethyl] -2,3-dihydro-1H-indol-2-one. As a continue research on the synthesis of indole-2,3-dione(isatin) derivatives, herein we report the synthesis and crystal structure of a new compound based on indoline-2,3-dione through the reaction of 1-(2-bromoethyl)-5-methylindoline-2,3-dione with 1-methylpiperazine, namely 5-methyl-3,3-bis(4-methylpiperazin-1-yl)-1-(2-(4-methylpiperazin-1-yl)ethyl) indolin-2-one.

In the title compound, each piperazine ring adopts a chair conformation. Two 1-methylpiperazine rings bond to one C8(sp^3^) of the pyrrole ring via the piperazine N atoms (N2, N6), while the third one links to the N1 atom of indolin-2-one moiety through a flexible ethyl group with an almost *syn* conformation (N1-C15-C16-N5 torsion angle of 58.0 (3)°, as shown in Fig. 1). This steric configuration is similar to that of 5-methyl-3,3-bis(morpholin-4-yl)-1-[2(morpholin-4-yl)ethyl] -2,3-dihydro-1H-indol-2-one (59.7 (3)°) reported by us recently (Lin *et al.*, 2012). Through C19—H19b(methylene)···O2^i^(carbonyl) interactions(see Table 1), the molecules are interconnected and arranged into an array along the *c* direction (i x, -y + 0.5, z +0.5), as shown in Fig. 2. The almost parallel arrays are stacked to form a three-dimensional framework (Fig. 3)

### Experimental

To a solution of 1-(2-bromoethyl)-5-methylindoline-2,3-dione (0.27 g, 1 mmol) in N,N-dimethylformamide (5 ml) was added dropwise 1-methylpiperazine (0.60 g, 6 mmol). The mixture was stirred at 80–90°C for 3 h. The colorless crystals of the title compound were deposited by evaporation of the resulting solution in room temperature for one day (m.p. 401.2–403.0 K; yield 45%).

### Refinement

All H atoms were discernible in the difference electron density maps. Nevertheless, the hydrogen atoms were placed into idealized positions and allowed to ride on their respective carrier atoms, with C—H = 0.93 and 0.97 Å for aryl and methylene hydrogens, respectively. *U*_iso_(H) = 1.2*U*_eq_(C)_aryl_/_methylene_.

### Crystal data

**Table:**

C_26_H_43_N_7_O	*F*(000) = 1024
*M**_r_* = 469.67	*D*_x_ = 1.145 Mg m^−^^3^
Monoclinic, *P*2_1_/*c*	Mo *K*α radiation, λ = 0.71073 Å
Hall symbol: -P 2ybc	Cell parameters from 233 reflections
*a* = 15.9433 (8) Å	θ = 2.2–27.0°
*b* = 14.4097 (6) Å	µ = 0.07 mm^−^^1^
*c* = 12.5310 (6) Å	*T* = 296 K
β = 108.774 (3)°	Block, colorless
*V* = 2725.7 (2) Å^3^	0.33 × 0.21 × 0.20 mm
*Z* = 4	

### Data collection

**Table:**

Bruker APEXII CCD area-detector diffractometer	4785 independent reflections
Radiation source: fine-focus sealed tube	3332 reflections with *I* > 2σ(*I*)
Graphite monochromator	*R*_int_ = 0.036
ω scans	θ_max_ = 25.0°, θ_min_ = 2.2°
Absorption correction: multi-scan (*SADABS*; Bruker, 2007)	*h* = −18→12
*T*_min_ = 0.613, *T*_max_ = 0.746	*k* = −15→17
17378 measured reflections	*l* = −14→14

### Refinement

**Table:**

Refinement on *F*^2^	Primary atom site location: structure-invariant direct methods
Least-squares matrix: full	Secondary atom site location: difference Fourier map
*R*[*F*^2^ > 2σ(*F*^2^)] = 0.047	Hydrogen site location: inferred from neighbouring sites
*wR*(*F*^2^) = 0.129	H-atom parameters constrained
*S* = 1.03	*w* = 1/[σ^2^(*F*_o_^2^) + (0.0638*P*)^2^ + 0.4418*P*] *P* = (*F*_o_^2^ + 2*F*_c_^2^)/3
4785 reflections	(Δ/σ)_max_ < 0.001
307 parameters	Δρ_max_ = 0.23 e Å^−^^3^
0 restraints	Δρ_min_ = −0.20 e Å^−^^3^

### Special details

**Table:**

Geometry. All esds (except the esd in the dihedral angle between two l.s. planes) are estimated using the full covariance matrix. The cell esds are taken into account individually in the estimation of esds in distances, angles and torsion angles; correlations between esds in cell parameters are only used when they are defined by crystal symmetry. An approximate (isotropic) treatment of cell esds is used for estimating esds involving l.s. planes.
Refinement. Refinement of F^2^ against ALL reflections. The weighted R-factor wR and goodness of fit S are based on F^2^, conventional R-factors R are based on F, with F set to zero for negative F^2^. The threshold expression of F^2^ > 2sigma(F^2^) is used only for calculating R-factors(gt) etc. and is not relevant to the choice of reflections for refinement. R-factors based on F^2^ are statistically about twice as large as those based on F, and R- factors based on ALL data will be even larger.

### Fractional atomic coordinates and isotropic or equivalent isotropic displacement parameters (Å^2^)

**Table:**

	*x*	*y*	*z*	*U*_iso_*/*U*_eq_	
O1	0.61856 (8)	0.42163 (9)	0.51428 (11)	0.0505 (4)	
N1	0.63105 (9)	0.48564 (10)	0.68622 (13)	0.0417 (4)	
N2	0.81475 (9)	0.38396 (9)	0.65785 (12)	0.0382 (4)	
N3	0.84094 (11)	0.18704 (11)	0.66137 (14)	0.0508 (4)	
N4	0.56630 (11)	0.10611 (11)	0.64539 (14)	0.0509 (4)	
N5	0.56530 (10)	0.29754 (10)	0.71078 (12)	0.0445 (4)	
N6	0.78671 (10)	0.52537 (10)	0.55620 (12)	0.0426 (4)	
N7	0.83904 (12)	0.65751 (12)	0.42139 (14)	0.0594 (5)	
C1	0.93336 (16)	0.62601 (16)	1.02535 (18)	0.0685 (7)	
H1A	0.9181	0.6508	1.0878	0.103*	
H1B	0.9600	0.6738	0.9936	0.103*	
H1C	0.9745	0.5758	1.0509	0.103*	
C2	0.85101 (14)	0.59104 (13)	0.93714 (16)	0.0508 (5)	
C3	0.76872 (15)	0.59731 (14)	0.95242 (18)	0.0567 (6)	
H3A	0.7651	0.6244	1.0182	0.068*	
C4	0.69174 (14)	0.56483 (13)	0.87348 (17)	0.0516 (5)	
H4A	0.6373	0.5694	0.8856	0.062*	
C5	0.69861 (12)	0.52560 (12)	0.77654 (15)	0.0411 (4)	
C6	0.77938 (12)	0.51941 (11)	0.75634 (15)	0.0385 (4)	
C7	0.85514 (13)	0.55156 (12)	0.83746 (15)	0.0457 (5)	
H7A	0.9095	0.5467	0.8253	0.055*	
C8	0.76536 (11)	0.47217 (12)	0.64355 (14)	0.0382 (4)	
C9	0.66284 (12)	0.45605 (12)	0.60356 (15)	0.0395 (4)	
C10	0.89282 (18)	0.10229 (16)	0.6758 (2)	0.0817 (8)	
H10A	0.8837	0.0659	0.7352	0.123*	
H10B	0.9545	0.1177	0.6947	0.123*	
H10C	0.8746	0.0674	0.6069	0.123*	
C14	0.80539 (13)	0.32577 (12)	0.74919 (16)	0.0457 (5)	
H14A	0.7441	0.3069	0.7319	0.055*	
H14B	0.8220	0.3611	0.8188	0.055*	
C12	0.79338 (14)	0.32871 (14)	0.55410 (16)	0.0529 (5)	
H12A	0.8012	0.3663	0.4938	0.064*	
H12B	0.7319	0.3092	0.5320	0.064*	
C13	0.86357 (14)	0.24095 (14)	0.76392 (16)	0.0529 (5)	
H13A	0.9251	0.2600	0.7847	0.063*	
H13B	0.8566	0.2029	0.8244	0.063*	
C11	0.85262 (15)	0.24454 (14)	0.57235 (17)	0.0575 (6)	
H11A	0.8384	0.2089	0.5031	0.069*	
H11B	0.9140	0.2641	0.5924	0.069*	
C21	0.61197 (17)	0.03109 (15)	0.6078 (2)	0.0715 (7)	
H21A	0.6241	−0.0186	0.6616	0.107*	
H21B	0.6666	0.0540	0.6012	0.107*	
H21C	0.5751	0.0087	0.5358	0.107*	
C19	0.62220 (14)	0.13991 (14)	0.75484 (17)	0.0523 (5)	
H19A	0.6796	0.1574	0.7500	0.063*	
H19B	0.6312	0.0905	0.8100	0.063*	
C20	0.58116 (14)	0.22236 (14)	0.79314 (16)	0.0536 (5)	
H20A	0.5256	0.2040	0.8030	0.064*	
H20B	0.6204	0.2439	0.8653	0.064*	
C18	0.54742 (15)	0.18231 (14)	0.56412 (17)	0.0555 (5)	
H18A	0.5075	0.1606	0.4925	0.067*	
H18B	0.6021	0.2019	0.5525	0.067*	
C17	0.50618 (13)	0.26400 (14)	0.60349 (16)	0.0526 (5)	
H17A	0.4948	0.3133	0.5479	0.063*	
H17B	0.4501	0.2456	0.6118	0.063*	
C16	0.53429 (13)	0.38064 (13)	0.75112 (18)	0.0516 (5)	
H16A	0.5688	0.3899	0.8297	0.062*	
H16B	0.4730	0.3717	0.7470	0.062*	
C15	0.54066 (12)	0.46790 (13)	0.68498 (17)	0.0498 (5)	
H15A	0.5025	0.4610	0.6076	0.060*	
H15B	0.5198	0.5207	0.7172	0.060*	
C22	0.73461 (14)	0.60954 (14)	0.51764 (18)	0.0550 (5)	
H22A	0.7543	0.6581	0.5738	0.066*	
H22B	0.6725	0.5973	0.5061	0.066*	
C25	0.87969 (13)	0.54758 (15)	0.57798 (18)	0.0558 (5)	
H25A	0.9160	0.4938	0.6086	0.067*	
H25B	0.8973	0.5975	0.6325	0.067*	
C26	0.8496 (2)	0.68403 (19)	0.3141 (2)	0.0871 (8)	
H26A	0.8137	0.7375	0.2846	0.131*	
H26B	0.8315	0.6336	0.2617	0.131*	
H26C	0.9107	0.6985	0.3254	0.131*	
C24	0.89265 (16)	0.57653 (16)	0.4690 (2)	0.0669 (6)	
H24A	0.9547	0.5908	0.4823	0.080*	
H24B	0.8764	0.5256	0.4156	0.080*	
C23	0.74686 (15)	0.63994 (15)	0.40848 (18)	0.0606 (6)	
H23A	0.7244	0.5921	0.3520	0.073*	
H23B	0.7128	0.6960	0.3821	0.073*	

### Atomic displacement parameters (Å^2^)

**Table:**

	*U*^11^	*U*^22^	*U*^33^	*U*^12^	*U*^13^	*U*^23^
O1	0.0457 (8)	0.0621 (8)	0.0380 (8)	−0.0015 (6)	0.0053 (6)	−0.0015 (7)
N1	0.0393 (8)	0.0425 (8)	0.0440 (9)	0.0020 (7)	0.0145 (7)	−0.0015 (7)
N2	0.0452 (9)	0.0371 (8)	0.0313 (8)	0.0021 (6)	0.0110 (7)	0.0003 (7)
N3	0.0609 (10)	0.0404 (9)	0.0488 (10)	0.0074 (8)	0.0145 (8)	−0.0025 (8)
N4	0.0605 (10)	0.0466 (9)	0.0479 (10)	−0.0058 (8)	0.0206 (8)	−0.0001 (8)
N5	0.0497 (9)	0.0447 (9)	0.0377 (9)	−0.0012 (7)	0.0122 (7)	0.0013 (8)
N6	0.0429 (9)	0.0427 (8)	0.0428 (9)	0.0008 (7)	0.0145 (7)	0.0079 (7)
N7	0.0748 (12)	0.0513 (10)	0.0556 (11)	−0.0103 (9)	0.0261 (10)	0.0090 (9)
C1	0.0831 (16)	0.0589 (13)	0.0493 (13)	−0.0106 (12)	0.0016 (12)	−0.0094 (11)
C2	0.0689 (14)	0.0371 (10)	0.0406 (11)	−0.0035 (9)	0.0097 (10)	−0.0022 (9)
C3	0.0830 (16)	0.0438 (11)	0.0445 (12)	−0.0027 (11)	0.0225 (12)	−0.0111 (10)
C4	0.0633 (13)	0.0445 (11)	0.0530 (12)	0.0023 (10)	0.0270 (11)	−0.0069 (10)
C5	0.0499 (11)	0.0331 (9)	0.0404 (11)	0.0029 (8)	0.0146 (9)	−0.0004 (8)
C6	0.0443 (10)	0.0337 (9)	0.0366 (10)	0.0010 (8)	0.0118 (8)	0.0000 (8)
C7	0.0499 (11)	0.0401 (10)	0.0442 (11)	−0.0021 (8)	0.0111 (9)	−0.0003 (9)
C8	0.0401 (10)	0.0388 (9)	0.0353 (10)	0.0005 (8)	0.0118 (8)	−0.0003 (8)
C9	0.0428 (10)	0.0382 (9)	0.0358 (10)	0.0028 (8)	0.0102 (9)	0.0049 (9)
C10	0.109 (2)	0.0567 (14)	0.0793 (18)	0.0317 (14)	0.0299 (16)	0.0022 (13)
C14	0.0561 (12)	0.0420 (10)	0.0409 (11)	0.0034 (9)	0.0182 (9)	0.0005 (9)
C12	0.0704 (14)	0.0486 (11)	0.0365 (11)	0.0082 (10)	0.0125 (10)	−0.0037 (10)
C13	0.0628 (13)	0.0489 (11)	0.0428 (11)	0.0087 (10)	0.0113 (10)	0.0053 (10)
C11	0.0729 (14)	0.0542 (12)	0.0475 (12)	0.0109 (11)	0.0224 (11)	−0.0067 (11)
C21	0.0855 (17)	0.0564 (13)	0.0788 (17)	−0.0015 (12)	0.0349 (14)	−0.0081 (13)
C19	0.0576 (12)	0.0496 (11)	0.0488 (12)	−0.0002 (10)	0.0160 (10)	0.0078 (10)
C20	0.0658 (13)	0.0564 (12)	0.0389 (11)	−0.0026 (10)	0.0175 (10)	0.0048 (10)
C18	0.0689 (14)	0.0559 (12)	0.0400 (11)	−0.0113 (11)	0.0152 (10)	−0.0015 (10)
C17	0.0530 (12)	0.0550 (12)	0.0439 (11)	−0.0058 (10)	0.0072 (10)	0.0039 (10)
C16	0.0478 (11)	0.0562 (12)	0.0568 (13)	−0.0028 (9)	0.0251 (10)	−0.0066 (11)
C15	0.0408 (11)	0.0527 (12)	0.0568 (12)	0.0077 (9)	0.0169 (9)	0.0004 (10)
C22	0.0590 (13)	0.0463 (11)	0.0610 (14)	0.0050 (10)	0.0210 (11)	0.0112 (11)
C25	0.0480 (12)	0.0617 (13)	0.0589 (13)	−0.0020 (10)	0.0189 (10)	0.0096 (11)
C26	0.121 (2)	0.0796 (18)	0.0699 (17)	−0.0179 (16)	0.0443 (16)	0.0146 (14)
C24	0.0666 (14)	0.0676 (14)	0.0772 (16)	−0.0018 (12)	0.0379 (13)	0.0128 (13)
C23	0.0716 (15)	0.0481 (12)	0.0570 (14)	−0.0014 (11)	0.0136 (11)	0.0137 (11)

### Geometric parameters (Å, º)

**Table:**

O1—C9	1.220 (2)	C14—H14A	0.9700
N1—C9	1.360 (2)	C14—H14B	0.9700
N1—C5	1.410 (2)	C12—C11	1.509 (3)
N1—C15	1.459 (2)	C12—H12A	0.9700
N2—C14	1.465 (2)	C12—H12B	0.9700
N2—C12	1.468 (2)	C13—H13A	0.9700
N2—C8	1.476 (2)	C13—H13B	0.9700
N3—C13	1.445 (2)	C11—H11A	0.9700
N3—C11	1.449 (3)	C11—H11B	0.9700
N3—C10	1.453 (3)	C21—H21A	0.9600
N4—C19	1.458 (3)	C21—H21B	0.9600
N4—C18	1.461 (2)	C21—H21C	0.9600
N4—C21	1.464 (3)	C19—C20	1.507 (3)
N5—C16	1.447 (2)	C19—H19A	0.9700
N5—C17	1.454 (2)	C19—H19B	0.9700
N5—C20	1.461 (2)	C20—H20A	0.9700
N6—C25	1.454 (2)	C20—H20B	0.9700
N6—C22	1.462 (2)	C18—C17	1.507 (3)
N6—C8	1.464 (2)	C18—H18A	0.9700
N7—C23	1.448 (3)	C18—H18B	0.9700
N7—C24	1.456 (3)	C17—H17A	0.9700
N7—C26	1.459 (3)	C17—H17B	0.9700
C1—C2	1.505 (3)	C16—C15	1.527 (3)
C1—H1A	0.9600	C16—H16A	0.9700
C1—H1B	0.9600	C16—H16B	0.9700
C1—H1C	0.9600	C15—H15A	0.9700
C2—C3	1.388 (3)	C15—H15B	0.9700
C2—C7	1.393 (3)	C22—C23	1.508 (3)
C3—C4	1.387 (3)	C22—H22A	0.9700
C3—H3A	0.9300	C22—H22B	0.9700
C4—C5	1.376 (3)	C25—C24	1.505 (3)
C4—H4A	0.9300	C25—H25A	0.9700
C5—C6	1.393 (2)	C25—H25B	0.9700
C6—C7	1.384 (2)	C26—H26A	0.9600
C6—C8	1.519 (2)	C26—H26B	0.9600
C7—H7A	0.9300	C26—H26C	0.9600
C8—C9	1.565 (2)	C24—H24A	0.9700
C10—H10A	0.9600	C24—H24B	0.9700
C10—H10B	0.9600	C23—H23A	0.9700
C10—H10C	0.9600	C23—H23B	0.9700
C14—C13	1.509 (3)		
			
C9—N1—C5	111.34 (14)	N3—C11—C12	110.32 (17)
C9—N1—C15	123.09 (16)	N3—C11—H11A	109.6
C5—N1—C15	125.23 (15)	C12—C11—H11A	109.6
C14—N2—C12	109.19 (14)	N3—C11—H11B	109.6
C14—N2—C8	113.78 (13)	C12—C11—H11B	109.6
C12—N2—C8	113.39 (13)	H11A—C11—H11B	108.1
C13—N3—C11	108.67 (15)	N4—C21—H21A	109.5
C13—N3—C10	111.86 (17)	N4—C21—H21B	109.5
C11—N3—C10	111.54 (17)	H21A—C21—H21B	109.5
C19—N4—C18	109.32 (15)	N4—C21—H21C	109.5
C19—N4—C21	109.44 (17)	H21A—C21—H21C	109.5
C18—N4—C21	110.04 (16)	H21B—C21—H21C	109.5
C16—N5—C17	113.91 (16)	N4—C19—C20	111.48 (17)
C16—N5—C20	111.57 (15)	N4—C19—H19A	109.3
C17—N5—C20	108.67 (15)	C20—C19—H19A	109.3
C25—N6—C22	108.43 (15)	N4—C19—H19B	109.3
C25—N6—C8	116.32 (14)	C20—C19—H19B	109.3
C22—N6—C8	115.95 (14)	H19A—C19—H19B	108.0
C23—N7—C24	110.28 (16)	N5—C20—C19	110.71 (16)
C23—N7—C26	110.92 (19)	N5—C20—H20A	109.5
C24—N7—C26	111.1 (2)	C19—C20—H20A	109.5
C2—C1—H1A	109.5	N5—C20—H20B	109.5
C2—C1—H1B	109.5	C19—C20—H20B	109.5
H1A—C1—H1B	109.5	H20A—C20—H20B	108.1
C2—C1—H1C	109.5	N4—C18—C17	111.87 (16)
H1A—C1—H1C	109.5	N4—C18—H18A	109.2
H1B—C1—H1C	109.5	C17—C18—H18A	109.2
C3—C2—C7	118.01 (18)	N4—C18—H18B	109.2
C3—C2—C1	121.10 (19)	C17—C18—H18B	109.2
C7—C2—C1	120.9 (2)	H18A—C18—H18B	107.9
C4—C3—C2	122.57 (19)	N5—C17—C18	109.94 (16)
C4—C3—H3A	118.7	N5—C17—H17A	109.7
C2—C3—H3A	118.7	C18—C17—H17A	109.7
C5—C4—C3	117.70 (19)	N5—C17—H17B	109.7
C5—C4—H4A	121.1	C18—C17—H17B	109.7
C3—C4—H4A	121.1	H17A—C17—H17B	108.2
C4—C5—C6	121.85 (18)	N5—C16—C15	113.69 (16)
C4—C5—N1	128.26 (17)	N5—C16—H16A	108.8
C6—C5—N1	109.88 (15)	C15—C16—H16A	108.8
C7—C6—C5	118.97 (17)	N5—C16—H16B	108.8
C7—C6—C8	131.72 (17)	C15—C16—H16B	108.8
C5—C6—C8	109.27 (15)	H16A—C16—H16B	107.7
C6—C7—C2	120.87 (18)	N1—C15—C16	112.12 (15)
C6—C7—H7A	119.6	N1—C15—H15A	109.2
C2—C7—H7A	119.6	C16—C15—H15A	109.2
N6—C8—N2	107.37 (13)	N1—C15—H15B	109.2
N6—C8—C6	117.46 (14)	C16—C15—H15B	109.2
N2—C8—C6	111.11 (14)	H15A—C15—H15B	107.9
N6—C8—C9	107.98 (13)	N6—C22—C23	108.37 (17)
N2—C8—C9	111.87 (14)	N6—C22—H22A	110.0
C6—C8—C9	100.97 (14)	C23—C22—H22A	110.0
O1—C9—N1	125.35 (17)	N6—C22—H22B	110.0
O1—C9—C8	126.21 (16)	C23—C22—H22B	110.0
N1—C9—C8	108.44 (15)	H22A—C22—H22B	108.4
N3—C10—H10A	109.5	N6—C25—C24	108.67 (17)
N3—C10—H10B	109.5	N6—C25—H25A	110.0
H10A—C10—H10B	109.5	C24—C25—H25A	110.0
N3—C10—H10C	109.5	N6—C25—H25B	110.0
H10A—C10—H10C	109.5	C24—C25—H25B	110.0
H10B—C10—H10C	109.5	H25A—C25—H25B	108.3
N2—C14—C13	110.23 (15)	N7—C26—H26A	109.5
N2—C14—H14A	109.6	N7—C26—H26B	109.5
C13—C14—H14A	109.6	H26A—C26—H26B	109.5
N2—C14—H14B	109.6	N7—C26—H26C	109.5
C13—C14—H14B	109.6	H26A—C26—H26C	109.5
H14A—C14—H14B	108.1	H26B—C26—H26C	109.5
N2—C12—C11	110.25 (16)	N7—C24—C25	111.09 (19)
N2—C12—H12A	109.6	N7—C24—H24A	109.4
C11—C12—H12A	109.6	C25—C24—H24A	109.4
N2—C12—H12B	109.6	N7—C24—H24B	109.4
C11—C12—H12B	109.6	C25—C24—H24B	109.4
H12A—C12—H12B	108.1	H24A—C24—H24B	108.0
N3—C13—C14	111.04 (16)	N7—C23—C22	111.87 (18)
N3—C13—H13A	109.4	N7—C23—H23A	109.2
C14—C13—H13A	109.4	C22—C23—H23A	109.2
N3—C13—H13B	109.4	N7—C23—H23B	109.2
C14—C13—H13B	109.4	C22—C23—H23B	109.2
H13A—C13—H13B	108.0	H23A—C23—H23B	107.9

### Hydrogen-bond geometry (Å, º)

**Table:**

*D*—H···*A*	*D*—H	H···*A*	*D*···*A*	*D*—H···*A*
C19—H19*B*···O1^i^	0.97	2.64	3.388 (2)	135

Symmetry code: (i) *x*, −*y*+1/2, *z*+1/2.
